# Oestrogen receptor negativity in breast cancer: a cause or consequence?

**DOI:** 10.1042/BSR20160228

**Published:** 2016-12-23

**Authors:** Vijaya Narasihma Reddy Gajulapalli, Vijaya Lakshmi Malisetty, Suresh Kumar Chitta, Bramanandam Manavathi

**Affiliations:** * Department of Biochemistry, Molecular and Cellular Oncology Laboratory, School of Life Sciences, University of Hyderabad, Hyderabad 500046, India; † Department of Biotechnology, Acharya Nagarjuna University, Guntur, Andhra Pradesh 522510, India; ‡ Department of Biochemistry, Sri Krishnadevaraya University, Anantapur, Andhra Pradesh 515002, India

**Keywords:** endocrine resistance, epigenetic factors, microRNAs (miRNAs), oestrogen receptor α, oestrogen receptor (ER)-negative breast cancer, ubiquitin ligases

## Abstract

Endocrine resistance, which occurs either by *de novo* or acquired route, is posing a major challenge in treating hormone-dependent breast cancers by endocrine therapies. The loss of oestrogen receptor α (ERα) expression is the vital cause of establishing endocrine resistance in this subtype. Understanding the mechanisms that determine the causes of this phenomenon are therefore essential to reduce the disease efficacy. But how we negate oestrogen receptor (ER) negativity and endocrine resistance in breast cancer is questionable. To answer that, two important approaches are considered: (1) understanding the cellular origin of heterogeneity and ER negativity in breast cancers and (2) characterization of molecular regulators of endocrine resistance. Breast tumours are heterogeneous in nature, having distinct molecular, cellular, histological and clinical behaviour. Recent advancements in perception of the heterogeneity of breast cancer revealed that the origin of a particular mammary tumour phenotype depends on the interactions between the cell of origin and driver genetic hits. On the other hand, histone deacetylases (HDACs), DNA methyltransferases (DNMTs), miRNAs and ubiquitin ligases emerged as vital molecular regulators of ER negativity in breast cancers. Restoring response to endocrine therapy through re-expression of ERα by modulating the expression of these molecular regulators is therefore considered as a relevant concept that can be implemented in treating ER-negative breast cancers. In this review, we will thoroughly discuss the underlying mechanisms for the loss of ERα expression and provide the future prospects for implementing the strategies to negate ER negativity in breast cancers.

## INTRODUCTION

Throughout the world, breast cancer remains as one of the prevailing malignancies affecting millions of women, although it is scarce in men. Despite of our increased understanding of the disease and the improved diagnosis, a large number of new cases are still being registered, challenging the current diagnostic measures. For instance, the estimated new breast cancer cases and deaths by Sex in United States for the year 2016 is 249260 and 40890 respectively [1]. Breast cancer can originate from different areas of the breast that include the ducts, lobules or in some cases, between the breasts. The majority of breast cancers originates from epithelial cells and hence are called ‘carcinomas’ [2]. When left untreated, breast cancer can metastasize to other areas of the body, preferably to bone, lung, liver or brain and can cause malignancies.

## BREAST CANCER CLASSIFICATION

**Figure 1:**
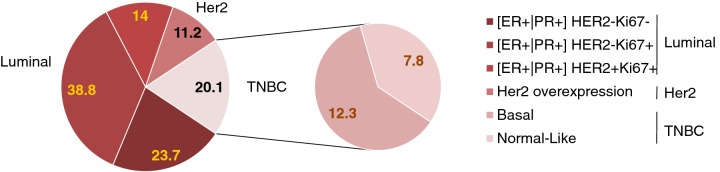
The breast cancer classification The pie diagram represents percentage of different molecular subtypes of breast cancers.

Breast cancer is heterogeneous in nature as it comprises various cell types with distinct biological features and clinical behaviour. Breast cancers are classified as invasive or non-invasive types on the basis of localization and the extent of the tumour spread [3]. On a molecular basis (gene expression profile), breast cancers are classified into the following major subtypes (Figure 1) [4–12]. Each of these tumours has different risk factors, for instance response to treatment, disease progression and preferential metastasis sites [13,14]. Further, the aetiology, pathogenesis, and prognosis of breast cancer in patients of various races/ethnicities are significantly influenced by intrinsic molecular breast cancer subtypes across the different populations around the globe [15]. PAM50 signature assay is by far the most recent classification of breast cancer by molecular approach techniques, which measures 50 genes quantitatively. This assay was developed by Parker et al. [16], for subclassification of breast cancers into three molecular subtypes [luminal A/B, basal-like (BL) and human epidermal growth factor receptor 2 (Her-2)]. The modern classification of breast cancer subtypes based on gene expression profiling of the tumours facilitated the clinical implications and the predictive values of each subtype. A recent report showed that the St. Gallen surrogate classification of breast cancer subtypes can successfully predicts tumour presenting features, nodal involvement, recurrence patterns and disease-free survival [17]. Further, intrinsic molecular profiling provides clinically relevant information endorsed by St. Gallen consensus panel [11]. In view of the heterogeneous nature of breast cancer, the optimal classification and subtyping of each tumour will eventually help in the development of a conspicuous therapy.

### Triple-negative breast cancer

Based on the immunohistochemical analysis, triple-negative breast cancers (TNBCs) have been identified as breast cancers that do not express oestrogen receptorα (ERα), progesterone receptor (PR) and Her-2 (triple-negative immunophenotype) [18]. Within the TNBCs, using gene expression and cluster analysis, Lehmann et al. [19] identified six subtypes that include two BL (BL1 and BL2), an immunomodulatory (IM), a mesenchymal (M), a mesenchymal stem-like (MSL) and a luminal androgen receptor (LAR) subtype. Previously, Prat et al. [20] subclassified TNBCs into BL (70%) or non-basal-like (NBL) breast cancers (approximately 25%) based on gene expression profiling data. Irrespective of these different classifications, basically all TNBCs are aggressive in nature and associated with more proliferation and metastasis than other subtypes. TNBCs account for up to 20% of all breast cancers. These types of tumours are associated with BRCA1 and BRCA2 mutations [21]. With respect to treatment, BL breast cancer patients within TNBC, but not in non-basal type, appear to benefit with either carboplatin or bevacizumab, an anti-vascular endothelial growth factor (VEGF) monoclonal antibody therapy in neoadjuvant setting [22]. On the other hand, the NBL (i.e. luminal A, luminal B and Her-2-enriched) or AR-positive, ER and PR-negative metastatic breast cancers might benefit from anti-androgens [23]. However, in many cases the option for treatment is chemotherapy only, as the TNBC tumours are not amenable to conventional targeted therapies [24].

### Her-2 positive breast cancers

Her-2 positive breast tumours are characterized by the lack of expression of luminal/ER-related genes and overexpression or augmentation of Her-2 genes associated with aggressive phenotypes. *ERBB2* gene encodes for a transmembrane tyrosine kinase receptor (Her-2) that belongs to the epidermal growth factor (EGFR) family. These tumours are frequently high-grade and 50% of them exhibit p53 mutations and are associated with poor prognosis [16,25]. This subtypes comprise approximately14% of all the breast tumours and can be effectively treated by various anti-Her-2 therapies such as trastuzumab or lapatinib [25].

### Luminal breast cancer

Approximately two-thirds of breast cancers are ER-positive [26–28] that are specified by the expression of ERα and PR in breast tumours. Because these tumours depend on oestrogen for their growth, treatment with selective oestrogen receptor modulators (SERMs) such as tamoxifen or raloxifene or aromatase, which are crucial for oestrogen biosynthesis, inhibitors like anastrozole or letrozole have better outcomes in these patients. However, many patients with ER-positive breast tumours fail to respond to endocrine therapy with tamoxifen, an anti-oestrogen, and most tumours that are initially responsive acquiring resistance by various mechanisms [29–31]. In recent years, high-throughput gene expression screening studies identify specific gene expression signatures that predict response to endocrine therapy and direct breast cancer patients for more appropriate therapeutic options [32,33]. In other studies, while using gene expression screening in mammary tumours, it was indicated that ER-positive breast tumours with poor response to endocrine therapy tend to have lower ERα expression and high levels of proliferation-associated genes [32,34–36]. Based on the proliferative index, luminal or ER-positive tumours were further classified into two intrinsic subtypes: luminal A and luminal B [37]. Luminal A breast cancers express high levels of ERα, lack of Her-2 expression, low expression of proliferative genes such as *Ki67* and low-grade (1 or 2). These tumours grow very slowly and have better prognosis than luminal B-type [38]. These tumours (luminal A) are successfully treated with endocrine therapy and have the best prognosis with high survival rates with low recurrence. On the other hand, low levels of ERα are expressed by luminal B tumours, which constitute approximately 10–20%, whereas Her-2 positive are often high-grade (2 or 3). Expression of proliferative markers like *Ki67* and cyclin B1 is higher in luminal B tumours than in luminal A. Tumours of this subgroup are associated with an unfavourable prognosis than in luminal A-type and may benefit from the chemotherapy [39]. They can be treated with targeted therapies, e.g. SERMs, such as tamoxifen or with aromatase inhibitors such as anastrozole in postmenopausal women [40].

## ER NEGATIVITY AND ENDOCRINE RESISTANCE IN BREAST CANCER

Anti-oestrogen resistance is likely to develop over time because of the highly pliable and adaptive nature of breast cancers to various selective pressures [41,42]. Anti-oestrogen resistance is of two types: *de novo* and acquired. The absence of both ERα and PR expressions represents the prevailing mechanisms of *de novo* resistance. However, approximately 25% of ER+/PR+, 66% of ER+/PR− and 55% of ER−/PR+ breast tumours do not respond to anti-oestrogens [42]. Several experimental studies suggest that loss of ERα can be due to long-term activation of growth factor signalling pathways. Approximately 30% of the patients display loss of ERα where EGFR/Her-2 activity is high [43,44], where the acquired resistance is defined by loss of anti-oestrogen responsiveness by initially responsive tumours. Most of the breast tumours initially responsive to anti-oestrogens confer acquired resistance [29], which express ERα at recurrence on anti-oestrogen therapy and are considered as ER+ tumours [45]. Although, tamoxifen has been shown to diminish the relapse and mortality rates of ER-positive breast cancers, a significant number of ER-positive tumours develop resistance to tamoxifen and become ER-negative [41]. It appears that a loss of ERα expression does not represent the major mechanism, driving acquired anti-oestrogen resistance. Furthermore, it is very difficult to attribute any single mechanism that confers anti-oestrogen resistance. Accumulating evidence suggests that several mechanisms acting at cellular or molecular levels are likely to be responsible for the endocrine resistance as discussed below.

Endocrine resistance is posing a major challenge today in treating significant percentage of breast cancers by hormone therapy. Understanding the mechanisms that underlie the causes of this phenomenon is therefore essential to reduce the burden of this disease. But how we negate ER negativity and endocrine resistance in breast cancers is questionable, to answer that two important approaches are considered: (1) understanding the origin of heterogeneity and ER negativity and (2) characterization of molecular regulators of endocrine resistance.

### Understanding the origin of heterogeneity and ER negativity

Breast cancers are heterogeneous anomalies having distinct molecular, cellular, histological and clinical behaviour [13]. Tumour heterogeneity is of two types: intra-tumour (within the tumour) and inter-tumour. Breast cancers exhibit both intra-tumour as well as inter-tumour heterogeneity. But the underlying biology causing tumour heterogeneity is yet to be fully understood. Due to the intra-tumour heterogeneity, breast cancer treatment has become more challenging today in clinical oncology studies [46]. To understand the tumour heterogeneity, it is essential to define the origin of each tumour cell type. Recent evidence suggests that the genetic lesions determine the tumour phenotype and cancers of distinct subtypes within a tissue, which may be derived from different ‘cells of origin’. Defined genetic alterations/changes may lead to the initiation of respective breast cancer cell type [47]. Although identification of cell-of-origin of each subtype of breast cancer is challenging, it would provide the identity and degree of transformation, which eventually enables us in better understanding of the breast tumour subtypes as well as it would help in predicting the tumour behaviour and early detection of malignancies. In normal breast cells where ER-positive cells rarely proliferate, whereas in breast tumours ER drives cell proliferation [48]. The lack of proliferation in the ER-positive ductal epithelium indicates a positive link between ERα expression and terminal differentiation in the normal breast cells and it further implies that ER-positive and -negative tumours arise from distinct cell types. Recent studies in model systems reported that luminal progenitors will serve as precursors for BL tumours if they receive a genetic or epigenetic event(s) that could change the phenotypes [49–53]. For instance, deletion of *BRCA1* or *PTEN* in luminal epithelial cells results in loss of luminal differentiation, and then oncogenic insults in these cells, leading to the formation of BL tumours [54].

Mouse models were used to address if the origin of a particular mammary tumour phenotype depends on the interactions between the cell of origin and driver genetic hits. Melchor et al. [55] generated mice deleted of Pten, p53, and BRCA2 in mammary basal epithelial cells or luminal ER-negative cells. Conditional deletion of BRCA2 and p53 in either basal or luminal ER-negative cells resulted in tumours with different latencies and histopathological features. For example, tumours in mice derived from p53, Pten or BRCA2 depletion in basal epithelial tumour cells displayed features of BL cells, whereas luminal ER-negative cell-origin tumours mimicked molecular subtypes of breast cancer, including BL and luminal B [55]. Transcriptome analysis from these tumours further provided the molecular link between the genetic lesion and tumour type. Consistent with the phenotypic data, gene expression signature of BRCA1:p53 mouse correlated with the human BL subtype and human BRCA1 breast cancers. The tumours of Pten deleted mice matched with the molecular features of luminal A and non-BRCA1/2 cancers, whereas *Brca2:p53/Pten:p53* gene signature had been seen across the range of human breast cancer molecular subtypes. Based on these observations, it has been concluded that initiating genetic lesion is the primary determinant of the molecular expression pattern of resulting tumours. Furthermore, the genetic lesions together with a cell of origin serve as strict drivers of tumour phenotype but not the cell of origin alone, reiterating the fact that mammary tumour heterogeneity is a result of interactions between the cell of origin and early genetic events.

The breast cancer can be initiated in a single cell by a combined effect of genetic and epigenetic events, suggesting that breast cancer is a monoclonal disease. Subsequent tumour progression is driven by the accumulation of additional genetic changes combined with clonal expansion and selection. The two models such as the cancer stem cell (CSC) and the clonal evolution and selection hypotheses agree that tumours originate from a single cell. However, controversies prevail regarding the tumour heterogeneity, progression and development of drug resistance. The differences between two models depict how a transformed cell acquires multiple mutations and unlimited proliferative potential. In particular, these two models explain tumour heterogeneity with different mechanisms: CSC suggests tumour heterogeneity as a programme of aberrant differentiation, whereas clonal evolution supports that it is a result of competition among tumour cells with different phenotypes [56,57].

Tamoxifen treatment and heterogeneity have an intimate association in the development of endocrine resistance in breast cancer. Many breast cancers that arise after tamoxifen treatment are typically ER-negative, although premalignant lesions such as atypical ductal hyperplasia are highly ER-positive. The p53 null mouse mammary epithelial transplant model is characterized by ER-positive premalignant lesions that give rise to both ER-positive and -negative tumours. Given this progression from ER-positive to ER-negative lesions, Medina et al. [58] tested the ability of tamoxifen to block or delay mammary tumorigenesis in several versions of this model. Tamoxifen blocked oestrogen signalling in these mice as evident by a decrease in progesterone-induced lateral branching and epithelial proliferation in the mammary epithelium. Tamoxifen also significantly delayed tumorigenesis in ER-positive high premalignant line PN8a from 100% to 75%. From the present study, the authors derive that tamoxifen delays the emergence of ER-negative tumours if given in early stages of premalignant progression [58].

Recently, attempts were made to generate a novel heterogeneous, spontaneous mammary tumour animal model of Kunming mice (*Mus musculus*, Km) which is ER-negative that have developed invasive ductal tumours that spread through the blood vessel into the liver and lungs. The mammary tumours are either ER- or PR-negative, whereas Her-2 protein is weakly positive. In addition, these tumours also had high expression of VEGF, moderate or high expression of c-Myc and cyclin D1 that elucidates that this is one of the first spontaneous mammary models displaying colony strain of outbred mice and could serve as a pivotal tool in understanding the biology of anti-hormonal breast cancer in women [59]. These mouse models can be further explored to study the origin of ER negativity and to further understand the endocrine resistance.

### Characterization of molecular regulators of endocrine resistance in breast cancer

**Figure 2:**
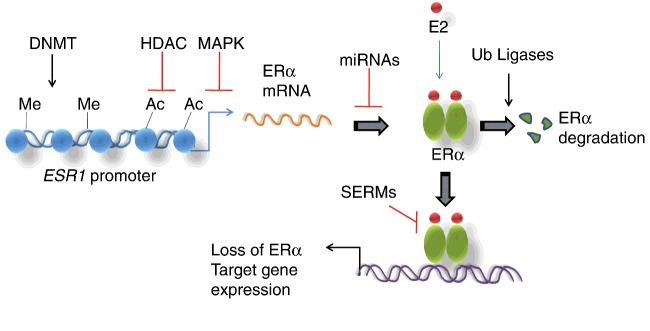
Pathways driving ER negativity and endocrine resistance in breast cancer Schematic representation of roles of various regulatory mechanisms in loss of ERα expression and function in ER-negative breast cancer. Epigenetic regulators such as DNMTs, HDACs and ER-specific miRNAs negatively regulate ERα expression. The ERα expression is also lost by hyperactive MAPK pathway. ER-specific ubiquitin ligases promote ERα degradation through ubiquination mechanism. These three types of molecular regulators ensure endocrine resistance in ER-negative breast cancer.

Because ERα is responsible for the development and progression of majority of breast cancers, current therapies target ERα functions where tamoxifen, an anti-oestrogen, has been the principal front-line therapy for breast cancers for the last three decades [60,61]. But a large number of patients displayed tamoxifen resistance posing a major challenge in treating these patients [36,62]. Although reduced expression of ERα is one of the major contributing factors to the endocrine resistance [63,64], the mechanism of ERα down-regulation in endocrine resistance is not fully understood. Recent advancements in the field suggest that epigenetic modifications, miRNA-mediated gene silencing and proteasomal degradation, either of which can cause loss of ERα expression resulting in ER negativity of breast cancers (Figure 2).

#### Epigenetic regulation of ERα and development of ER negativity in breast cancer

Mammalian genomes contain a high degree of punctuated DNA sequences of CpG called CpG islands [65]. Methylation of DNA at these CpG sites in the proximal regions of gene promoters is quite often linked to suppression of the respective gene expression [66], which is an epigenetic mechanism in which methyl groups are covalently attached to the 5′-carbon of a cytosine ring in a CpG-dinucleotide. Although CpG island methylation occurs in normal developmental processes such as X-chromosome inactivation and genomic imprinting, these CpG islands are usually not methylated in normal cells [67].

Methylation of the ERαgene promoter is intimately linked to loss of ERα expression in breast cancers [68]. Re-expression of ERα upon treatment of MDA-MB231 cells, an ER-negative breast cancer cell line, with 5-azacytidine, a DNA methyltransferase (DNMT) inhibitor, provided initial clues about the role of DNA methylation (Me) on ERα expression [69]. Indeed, this was further supported by the observation that ER-negative tumours maintained the methylation status of *ESR1* gene (encodes ERα) promoter, but not in ER-positive tumours implying that Me is the potential contributing factor for ER negativity in breast cancers [70]. Yan et al. [71] showed that DNMT1 is responsible for *ESR1* promoter methylation in ER-negative breast cancer cell lines, MDA-MB231. When DNMT1 expression was silenced by antisense oligonucleotides, the expression of ERα was retained in MDA-MB231 cells. Increased total DNMT activity and elevated levels of DNMT3B in a set of ER-negative cell lines as compared with ER positive cell lines further attributed to higher rates of methylation on promoters of *ESR1* in ER-negative cells [72]. In other studies, methyl-CpG-binding protein 2 (MeCP2) was shown to stabilize the methylation status of the *ESR1* gene promoter [73]. The MeCP2 is a component of nucleosome remodelling and deacetylase (NuRD) complex, which is a large protein complex containing the dual core histone deacetylases (HDAC) 1 and 2 (HDAC1 and 2), the metastasis-associated (MTA) proteins MTA1 (or MTA2/MTA3), the methyl-CpG-binding domain protein MBD3 or MeCP2, the chromodomain-helicase-DNA-binding protein CHD3 (Mi-2α) or CHD4 (Mi-2β) and the histone-binding proteins RbAp46 and RbAp48. As the Mi-2/NuRD complex contains deacetylase activity, MeCP2–NuRD complex represses ER expression by a dual mechanism involving methylation and deacetylation of *ESR1* promoter. Similarly, silencing of MTA1, another component of NuRD complex, is also shown to reduce the ERα expression in ER-positive breast cancer cells [74]. Binding of the NuRD complex to the ERα-target gene promoters has also been observed in ER-negative breast cancer cells re-expressing functional ERα in response to tamoxifen [75]. In contrast with these observations, a recent study postulated that an increased ERα expression in ERα-negative cells also increased its expression in ER-positive cells upon MTA1 silencing, differential recruitment of MTA1 transcriptional complex bound to ER promoter has been identified as the underlying mechanism causing it [76]. The transcriptional factors AP-2γ (TFAP2C) and the IFN-γ-inducible protein 16 (IFI16) were associated with MTA1 complex in MCF7 cells, in which TFAP2C activated *ESR1* gene transcription in contrast with MDA-MB231 cells where MTA1 complexed with IFI16 repressed the promoter activity and silenced the MTA1 that increased the expression of ERα [76]. In another study, a different model of epigenetic regulation of the *ESR1* promoter was proposed based on the experimental evidence obtained from ER-postive and -negative cell lines. In this model, an activator complex composed of pRb2/E2F4/5/HDAC1/SUV39H1/p300 binds to E2F boxes in the promoter region of *ESR1* gene. However the presence of p300, a HAT, overcomes the repressor activity imposed by both HDAC1 and the HMT SUV39H1 on *ESR1* promoter. Whereas in MDA-MB231 cells, methylation of CpG by DNMT3a/3b on this promoter induces the recruitment of ICBP90 [inverted CAAT box-binding protein (CBP) of 90 kDa] and consequently facilitate the replacement of p300 by DNMT1 in the repressor complex pRb2/E2F4/5/HDAC1/SUV39H1/DNMT1 to silence the *ESR1* gene expression [77]. Subsequently, MeCP2 is recruited to the methylated *ESR1* promoter to ensure its complete repression [78] that infers that distinct protein complexes with opposing transcriptional activities contribute to the epigenetic regulation of *ESR1* gene expression in different breast cancer cells. Similarly, inhibition of EZH2, a histone H3 Lys^27^ (H2K27) methyltransferase and polycomb group protein, is associated with up-regulation of ERα in breast cancer cells, suggesting that targeting of EZH2 provides an option for restoring response to tamoxifen in endocrine-resistant breast cancers [79]. In addition to these intrinsic regulators, arsenic also has been shown to induce re-expression of functional ERα in MDA-MB231 cells [80]. The re-expression of ERα by arsenic involves repression of *DNMT1* and *DNMT3a* expression along with partial dissociation of DNMT1 protein from the *ESR1* promoter in these cells. Thus, it can be concluded that *ESR1* promoter is under constant threat from the protein complexes that contain methylation and deacetylation enzymes and, provides an option to target these mechanisms to re-express ERα that eventually restores the hormone sensitivity and response to endocrine therapy in ER-negative breast cancers.

Attempts were made to test the therapeutic effects of methylation and deacetylation inhibitors both *in vitro* and *in vivo*. Zhou et al. [81] showed that treatment with HDAC inhibitor suberoylanilide hydroxamic acid (SAHA) resulted in the re-expression of ERα coupled with the loss of EGFR in ER-negative MDA-MB231 cells and restored tamoxifen sensitivity in these cells. Down-regulation of EGFR by SAHA is due to the attenuation of its mRNA stability. In contrary, Yi et al. [82] reported that SAHA enhances ERα degradation through C-terminus of Hsp70-interacting protein (CHIP)-mediated proteasomal pathway in MCF7 cells, an ER-positive breast cancer cell line and thus can be postulated that opposing effects of SAHA in different breast cancer cells could be due to the cell lines used, however precise mechanisms are yet to be identified. The combined therapy using both DNMT and HDAC inhibitors displays better assurance to treat ER-negative breast cancers [83]. Valproic acid (VPA), an HDAC inhibitor, is also shown to restore oestrogen sensitivity in MDA-MB231 cells by inducing the re-expression of ERα and FoxA1, a co-activator of ERα [84]. Another study showed that letrozole treatment in combination with entinostatin, an HDAC inhibitor, increased the sensitivity in xenografts where letrozole alone had significant reduction in the expression of ERα but there was a marked increase in the expression of Her-2 also [85]. As growth factor signalling antagonizes ERα expression, treating it with trastuzumab (anti-Her-2 antibody) ablates Her-2 action, leading to increased expression of ERα and enhances its sensitivity to endocrine therapy [86,87]. However, the exact mechanism of trastuzumab blocking Her-2 leading to up-regulation of ERα remains elusive. A recent study shows that trastuzumab treatment enhances Myc–SMRT interactions in Her-2 overexpressing breast cancer cells and inhibits expression of the Myc target gene, *survivin* [88]. Further trastuzumab treatment induces the interaction between CBP and ERα which in turn enhances ERα transcriptional activity and expression of the ERα target gene, *pS2*. Furthermore, metastatic tissues from patients who had failed for trastuzumab therapy were pS2-positive providing the proof that trastuzumab treatment can benefit endocrine-resistant breast cancer patients with hormone therapy [88]. Recent studies also showed that FTY720 and avermectin, inhibitors of HDAC and SIN3 corepressor, as a novel strategy to restore tamoxifen sensitivity in ER-negative and TNBC tumours [89,90]. Overall, these studies showed the combination therapy using various inhibitors of epigenetic modulators provide a new arsenal to the limited list of therapies to endocrine-resistant breast cancer treatments.

#### Role of miRNAs in the development of ER negativity in breast cancer

miRNAs are small non-coding RNA molecules with a length of 18––22 nucleotides, miRNAs are naturally synthesized by mammalian cells that mostly are evolutionary conserved. These small RNAs modulate post-transcriptional expression of protein-coding genes in diverse biological processes including cell cycle, survival, differentiation, autophagy and senescence [91,92]. miRNAs bind to 3′-UTR of mRNA transcripts and inhibit their translation either by degradation or destabilization of target mRNA [93]. Large data suggest that dysregulated expression of miRNAs is found in many cancers, including breast cancer [94–97].

The connection between miRNAs and breast cancers was derived from studies investigating the expression of miRNAs in breast cancer cell lines and tumour samples. As 3′-UTR of *ERα* mRNA, which is approximately 4.3 kb long, contains several putative binding sites for various miRNAs created curiosity to investigate the role of miRNAs on ERα functions and its functional relevance to breast cancer development. *miR-206* was the first miRNA reported to regulate ERα expression in breast cancer cells, *miR-206* has two binding sites within the 1200 bp region in the 3′-UTR of ERα. Overexpression of *miR-206* in MCF7 cells led to the decrease in ERα levels, but has no effect on ERβ and the expression levels of ERα target genes such as *PR*, *CCDN1* and *pS2* [98]. Similar to *miR-206*, *miR-221* and *miR-222* levels that are elevated in ER-negative breast cancers could decrease ERα protein levels by binding to 3′-UTR of ERα. *miR-221/222* expression confers tamoxifen and fulvestrant resistance in ER-positive breast cancer cells indirectly contributing to ER negativity [99,100]. It appears that *miR-221/222* expression confers fulvestrant resistance by activating β-catenin and modulating TGF-β and p53 signalling [101]. Further, elevated levels of *miR-221/222* were found in ER-negative and Her-2-positive breast cancer cells. Silencing of these two miRNAs partially restores ERα protein expression, tamoxifen-induced cell growth arrest and apoptosis. In contrast, ectopic expression of *miR-221/222* in ER-positive cells reduced levels of ERα and conferred resistance to tamoxifen [63,102]. In another study, *miR-22* was identified as a potential ERα-targeting miRNAs [103]. Ectopic expression of *miR-22* caused degradation of *ERα* mRNA and inhibition of ERα-dependent proliferation of breast cancer cells. Further, *miR-22* expression was found to be down-regulated in ER-positive human breast cancer cell lines and tumour specimens [103,104]. High level expression of *miR-22* in MDA-MB231 decreased ERα levels and subsequently induced apoptosis. Let-7 is an ERα targeting miRNA whose expression is low in ER-positive breast cancer cell lines. Studies by Zhao et al. [105] revealed that ectopic expression of let-7 miRNA in MCF7 cells decreases ERα activity and cell proliferation, and subsequently induces apoptosis in MCF7 cells. Furthermore, let-7 expression was inversely correlated with invasion and metastasis, which indicates that loss of ER expression by let-7 may result in poor clinical outcomes and resistance to endocrine therapy [106]. Since the activity of co-regulators is crucial for ERα functioning, miRNAs that target co-regulators could also indirectly influence the functionality of ERα in breast cancer cells. Consistent with this notion, *miR-17-5p*, represses the AIB1/SRC-3, a co-activator of ERα, thereby attenuating ERα-mediated cell proliferation [107]. Expression of *miR-17-5p* was low in breast cancer cell lines. Hossain et al. [107] found that down-regulation of AIB1 by *miR-17-5p* results in decreased ERα target gene expression and proliferation of breast cancer cells.

In addition, high-throughput analysis of miRNAs expression in breast cancers brings about the prognostic value of breast cancer status irrespective of the influence of oestrogen on their expression and whether these miRNAs target ERα or not. For example, a microarray-based study identified that ERα is a target of miRNAs, *miR-18a/b*, *miR-193b*, *miR-206* and *miR-302c* [108]. Furthermore, high expression levels of *miR-18a* and *miR-18b* were correlated with ER-negative status in breast tumours [109]. Another recent study found that 20 miRNAs were significantly dysregulated in ER-positive compared with ER-negative breast cancers [109]. Of which, 12 miRNAs are up-regulated and eight are down-regulated. In particular, an *miR-190b* expression is found to be 23-fold higher in ER-positive as compared with ER-negative breast tumours [109]. Although the *miR-190b* expression is high in ER-positive breast tumours, its expression is not directly influenced by oestrogen and does not affect breast cancer cell proliferation.

In order to identify the miRNA-mediated tamoxifen resistance in breast cancers, Miller et al. [102] performed microarray studies comparing the miRNA profiles in tamoxifen-resistant compared with tamoxifen-sensitive MCF7 breast cancer cell lines [102] that revealed that eight miRNAs were significantly up-regulated whereas seven miRNAs were markedly down-regulated in tamoxifen-resistant MCF7 breast cancer cells as compared with tamoxifen-sensitive cells. Reintroduction of low expressing miRNAs in tamoxifen-resistant breast cancer cell lines could restore tamoxifen sensitivity. For instance, down-regulation of *miR-342* in Her-2-positive and -negative cell lines as well as in tamoxifen refractory breast tumours was found to be sensitive to tamoxifen when the expression of *miR-342* was restored. Hence, restoring *miR-342* expression could be a novel approach to sensitize refractory breast tumours to endocrine therapy [110,111]. Together, these studies imply that miRNAs those target ERα, contribute to the ER negativity in breast cancers and therefore, serve as potent therapeutic markers as well as targets in endocrine-resistant breast cancers (Table 1). Additional studies are required to confirm the roles of miRNAs in a clinical setting to get clear results. For clinical applications, miRNA expressions should be carefully validated prior to being adopted.

**Table 1:** The effect of various miRNAs on ERα expression and the breast cancer phenotype

Name of miRNA	miRNA function	Phenotype (breast cancer)	References
*miR-22*	ERα levels decreased	ERα-negative	[103,104]
*miR-206*	ERα levels decreased	ERα-negative	[98]
*miR-221*	ERα levels decreased	ERα-negative	[99,100]
*miR-222*	ERα levels decreased	ERα-negative	[99,100]
Let-7	ERα levels decreased	ERα-negative	[105]
*miR-193b*	ERα levels decreased	ERα-negative	[108]
*miR-190b*	ERα levels high	ERα-positive	[109]
*miR-302c*	ERα levels decreased	ERα-negative	[108]
*miR-342*	Sensitive to tamoxifen	ERα-positive	[110,111]
*miR-17-5p*	Represses AIB1/SRC-3 (ERα co-activator)	ERα-negative	[107]

#### Role of ubiquitination on ERα stability and breast cancer phenotype

The cellular levels of crucial regulators like kinases, receptors, phosphatases, transcription factors etc. are tightly regulated as their persistent high expression may have undesirable effects on the cell. Ciechanover et al. [112] first reported the selective degradation of protein through the conjugation of ubiquitin molecules in an ATP-dependent manner. Ubiquitinated proteins are recognized and degraded by the multi-subunit complex called the 26S proteasome [113]. This ubiquitin–proteasome pathway has a role in diverse cellular processing such as cell-cycle regulation, cell proliferation differentiation, apoptosis etc. in higher eukaryotes. Depending on the number of ubiquitins added to the target protein, ubiquitination is of two types: monoubiquitination and polyubiquitination. Although monoubiquitination is associated with diverse processes ranging from membrane transport to transcriptional regulation, polyubiquitination is mainly known to regulate protein turnover through proteasome-mediated degradation [114].

The first report about ER ubiquitination was investigated by Nirmala and Thampan [115]. They identified that the ERα in the uterus is ubiquitinated and this ubiquitination is enhanced by oestradiol treatment. The half-life of ERα in the presence of oestrogen is approximately 3–4 h [115] that was further supported by Nawaz et al. [116] depicted that ubiquitin-activating enzyme (UBA) and ubiquitin-conjugating enzymes (UBCs), can degrade ER protein *in vitro*. Treatment of cells with the proteasome inhibitor MG132 or lactacystin could significantly enhance the stability of ERα [116]. Subsequent studies clearly established that ERα undergoes ubiquitination upon ligand binding and this modification is important for efficient transactivation by the receptor [117]. Other than natural ligand, anti-oestrogen ICI-182,780 can induce proteasome-dependent proteolysis of ERα and therefore considered as a therapeutic drug for treating ER-positive breast cancers [118].

Many ubiquitin ligases are known to directly interact with ERα and stimulate its degradation and associate with breast cancer phenotype [119]. Fan et al. [120] identified that the CHIP, a chaperone-dependent E3 ligase, interacts directly with ERα and promotes ERα degradation through ubiquitination-proteasomal degradation pathway. The U-box (containing ubiquitin ligase activity) and the tetratricopeptide repeat (TPR, essential for chaperone binding) domains of CHIP are necessary for CHIP-mediated ERα degradation. Ectopic expression of the CHIP, resulted in decreased levels of endogenous ERα protein and impairment of ERα-mediated gene expression and hormone responsiveness in ER-positive cells. Notably, *PES1*, an oestrogen-inducible gene, inhibits CHIP-mediated ERα degradation mediated by CHIP. In contrast, *PES1* promotes CHIP-mediated ERβ ubiquitination and degradation. This differential regulation of ER protein stability lies in the interaction of PES1 with AF1 domain of ERα but not with ERβ. PES1 expression displayed good clinical outcome in breast cancers [121]. Whereas SAHA, an HDAC inhibitor, was reported to enhance ERα degradation through a CHIP-mediated proteasomal pathway in breast cancer MCF7 cells, suggesting the positive cross-talk between CHIP and SAHA in ER-positive breast cancers [82]. Von Hippel–Lindau (VHL), another E3 Ub ligase and a tumour suppressor, also regulates ERα stability. Ectopic expression of pVHL suppresses endogenous ERα levels and also promotes ubiquitination-mediated degradation of ERα [122]. pVHL-mediated ERα suppression is critical for the maintenance of microtubule organizing center (MTOC) as elevated ERα promotes MTOC amplification through disruption of BRCA1–Rad51 interaction and induces γ-tubulin expression [123]. Furthermore, activation of ERα signalling can increase γ-tubulin, a core factor of TuRC that renders resistance to taxol in breast tumours. Together, these findings suggest that pVHL-mediated ERα suppression is important for regulation of MTOC as well as drug resistance in breast tumours [123]. The speckle-type POZ protein (SPOP), an adaptor of Cullin3-based E3 ubiquitin ligase, also binds to ERα and targets ERα for ubiquitination-dependent degradation [124].

Neural precursor cell developmentally expressed down-regulation of 8 (NEDD8)–Uba3 pathway, which is shown to mediate ERα proteolysis [125]. Uba3 interacts with ligand-bound ERα through NR boxes that are important for the interaction between co-regulators and steroid hormone receptors. Uba3 has neddylation activity, which is required for inhibition of steroid receptor transactivation [126]. Duong et al. [127] reported that Mdm2, an oncogenic E3 ubiquitin-ligase, directly interacts with ERα in a ternary complex involving p53. This complex regulates both ligand-dependent and -independent reduction in ERα stability in human breast cancer cell lines, MCF7 [127]. Recent findings by Pan and colleagues showed that CUE domain containing protein CUEDC2 could promote ERα degradation through the ubiquitin-proteasome pathway [128]. By studying specimens from a large cohort of subjects with breast cancers, the authors found a strong inverse correlation between CUEDC2 and ERα expression. Notably, patients with high levels of CUEDC2 expression had poor responsiveness to tamoxifen treatment and high potential for relapse. Further, ectopic CUEDC2 expression impaired the responsiveness of breast cancer cells to tamoxifen, implying that CUEDC2 can contribute to resistance in breast cancer.

Not only the polyubiquitination but monoubiquitination of ERα has been associated with its functional activity. For instance, Lys^302^ of ERα is subjected to monoubiquitination by BRCA1/BARD1E3 Ub ligase [129]. Down-regulation of BRCA1 leads to activation of ERα, conversely ectopic expression of BRCA1 down-regulates ERα activity [130]. In contrary, monoubiquitination at Lys^302^ and Lys^303^ is shown to be important for ERα transcriptional activity and oestrogen-induced cell proliferation [131]. RNF31, an atypical E3 ubiquitin ligase, is also shown to monoubiquitinate ERα and increases ERα stability. This is consistent with the previous reports supporting the stabilization of ERα by its monoubiquitination. RNF31 and ERα association mainly occurs in the cytosol and activates the non-genomic mechanism, by which RNF31 via stabilizing ERα levels, controls the transcription of oestrogen-dependent genes linked to breast cancer cell proliferation [132]. Other than ubiquitination, ERα phosphorylation is also prone to proteasomal degradation and breast cancer phenotype. For instance, mitogen-activated protein kinase (p38MAPK)-mediated phosphorylation of ERα at Ser^294^ is prone to its turnover via the SCF (Skp2) proteasome-mediated pathway. Surprisingly, inhibition of p38MAPK or Skp2 knockdown restored functional ERα protein levels in ERα-negative breast cancer cells that suggests that p38MAPK or Skp2 is responsible for the loss of ER protein expression in ER-negative breast cancer cells [133].

**Figure 3:**
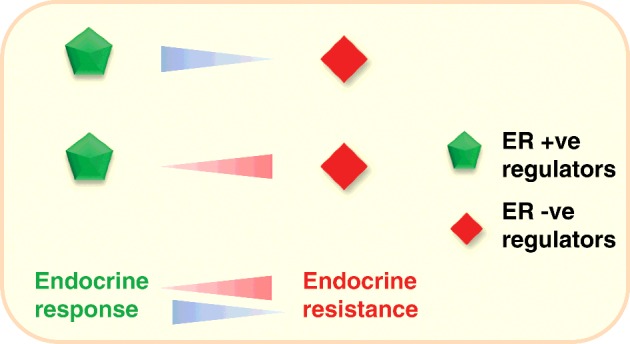
The relationship between endocrine resistance and ER regulators Schematic representation of a model depicting the subtle balance between ER regulators (+/–) dictate ER negativity and therefore endocrine resistance in breast cancer.

**Figure 4:**
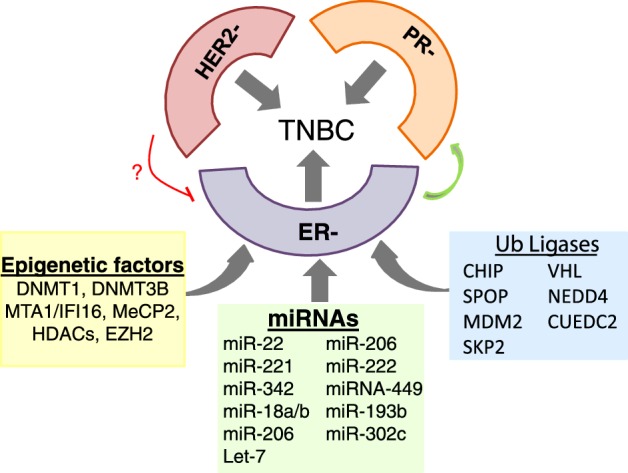
Pathways/factors driving triple-negative breast cancer Schematic representation of a model depicting the role of miRNAs, epigenetic factors and ubiquitin ligases that directly or indirectly regulate ERα expression and cause ER negativity and endocrine resistance in breast cancer. The ER negativity along with PR and Her-2 negativity together contribute to TNBC phenotype. As PR expression is dependent on ERα, loss of ERα expression leads to PR negativity. Because growth factor signalling antagonizes ERα expression, Her-2 negativity may lead to re-expression of ERα. But whether Her-2 negativity opposes ER negativity in breast cancer is unknown.

Over a decade of research on these aspects revealed that ERα regulators such as epigenetic factors and ubiquitin ligases emerged as vital contributors of ER negativity in breast cancers. The optimal balance between the expression of these regulators may predict the outcome of the endocrine response in breast cancer (Figure 3). With these data, we propose a model wherein various epigenetic factors and ubiquitin ligases directly or indirectly contribute to ER negativity and endocrine resistance in breast cancers by inhibiting ERα expression/functionality. The ER negativity along with PR and Her-2 negativity together contribute to TNBC phenotype. As oestrogen signalling via the ERα has been shown to up-regulate the expression of the *PR* gene and thus the majority of ER-positive tumours are also PR-positive. Therefore, loss of ERα expression could lead to PR negativity. Since Her-2 overexpression or amplification is associated with loss of ERα expression and vice versa, its overexpression is also a potential mechanism for ER negativity in breast cancer (Figure 4).

## ERα RESCUE THERAPY

The percentages of breast cancer cells, which become ER-negative that are initially ER-positive are not very high (10%) [134]. Due to acquired resistance, initially sensitive ERα+ breast cancers response to a second and even third line therapies falls with increasing lines of treatment [135]. It implies that the selective growth of ER-negative populations is not a common contributor to acquired resistance. However, it is difficult to assess whether ERα+ breast cancers that do not respond will become ER-negative with treatment or not. But this could be due to either the loss of ERα functionality or cells that might have lost their dependence on ERα to drive proliferation, and so the presence of functional ERα is no longer a requirement for cell survival and proliferation [41]. On the other hand, tumours that exhibit *de novo* resistance had an association between lower ERα expression to a lesser extent and lower rate of response to endocrine therapy [136]. This raised the possibility that re-expression of ERαmay benefit the endocrine therapy in these patients, but not in those who had tumours with acquired resistance.

Rescue therapy, also known as salvage therapy, is a form of therapy given to the patients who do not respond to the standard therapy. As the effects of anti-oestrogens such as tamoxifen are primarily mediated through the ERα, breast tumours expressing the receptor respond well to SERM therapy. However, approximately 30% of invasive breast cancers are hormone-independent because they lack ERα expression due to inactive *ESR1* promoter [137]. Many of the tumours that initially respond to tamoxifen can acquire resistance during and after tamoxifen therapy [30]. Therefore, ER negativity in breast carcinomas confronts to treat with anti-oestrogens. A hypothesis was emerged where re-expression of the ERα could restore the endocrine response in ER-negative cells. When ERα was ectopically expressed in an ER-negative breast cancer cell line (MDA-MB231), 17-β-oestradiol inhibited the proliferation of these cells, whereas the anti-oestrogens ICI182780 and tamoxifen blocked this effect indicating that ERα re-expression restores tamoxifen sensitivity in ER-negative cells [138]. Later on, several investigations led to provide the cross-talk between ERα expression and growth factor signalling [139,140]. Analysis of breast tumours using phospho-specific growth factor receptor antibodies revealed that erbB-2/Her-2 overexpressing tumours are ER/PR-negative [141], indicating that increased Her-2 receptor is associated with the ER-negative phenotype. Because ER-negative tumours often display overexpression or amplification of growth factor receptors of the erbB family, particularly EGFR and erbB-2, and consequently, elevated growth factor signalling and resultant MAP kinase (ERK) activity, EGFR or Her-2 overexpression in ER-positive breast cancer cells was investigated. Accordingly, overexpression of either EGFR or Her-2 in MCF7 cells results in acquisition of oestrogen-independence due to loss of ERα expression further supporting the fact that growth factor signalling and ERα expression have mutual inhibitory action on breast cancer cells [142,143]. Since MAPK is the downstream molecule of these growth factor signalling pathways, inhibition of this hyperactive MAPK restores ERα and acquired anti-oestrogen response [144,145]. An exception to this relationship is that hyperactivation of MAPK does not lead to re-expression of ERα in SUM-102 and SUM-159, two ER-negative basal type breast cancer cell lines that are found to exhibit hypermethylation of the *ESR1* promoter suggesting that additional mechanisms may operate to repress ERα expression in these cell lines [44]. Summing these studies, it can be concluded that the re-expression of ERα in ER-negative breast cancer cells by inhibiting EGFR or Her-2 signalling restores, at least in part, a hormone-responsiveness and could be useful as a potential therapeutic approach to endocrine-resistant breast cancer.

Initial studies on ER-negative breast cancer cells by treating with demethylating agents and HDAC inhibitors led to the expression of *ER* mRNA and functional protein. Fan et al. [146] reported that ERα can be re-expressed in ER-negative breast cancer cells by both DNMT1 inhibitor 5-aza-2'-deoxycytidine (AZA) and HDAC inhibitors, trichostatin A (TSA) and SAHA1. Another study by Zhou et al. [147] showed that ERα reactivation can be achieved using clinically relevant HDAC inhibitor LBH589 without demethylation of the CpG island within the *ESR1* promoter. These studies provide evidence that ER-negative breast cancer cells can be sensitized with anti-tumour effects of tamoxifen by combining treatment with 5-aza-dC/TSA. As indicated earlier, inhibition of growth factor signalling by trastuzumab that blocks Her-2/MAPK activation renders ERα re-expression and acquires the tamoxifen sensitivity. These studies provide new treatment options for patients with *de novo* resistance to endocrine therapies.

ERα re-expression is a win-win strategy to combat ER-negative breast cancer (personal opinion). Because the application of HDAC, DNMT or MEK inhibitors restores ERα expression in ER-negative breast cancer cells, these cells have responded to selective ERα antagonists [144,146]. However, studies by Bayliss et al. [44] demonstrated that ERα re-expression does not always result in effective responses to SERM therapy, which is because certain cancer cells fail to re-express ERα upon inhibition of the growth factor pathway. Over a period of time, the heterogeneity of a tumour might have changed due to which these tumour cells did not re-express ERα. Moreover, the systemic factors that account for establishing the local ecosystem within the tumour had opposed the re-expression of ERα. It implies that although combined therapy using these inhibitors along with tamoxifen has shown promising results *in vitro* and *in vivo* models, the following concerns need to be fully addressed before implementation of re-expression of ER therapy in clinics: (1) do all tumour cells respond to anti-oestrogens? In case of tumours that exhibit acquired resistance have developed more heterogeneity and may respond poorly to anti-oestrogens, (2) re-expression of ERα in those tumours with the application of HDAC, DNMT or MEK inhibitors may develop resistance to these inhibitors, (3) since ER-positive breast cancer cells die without ERα and ER-negative breast cancer copes without the receptor, why does one want to give another selective advantage to these tumour cells? and (4) because breast cancer cells will also gain the proliferative advantage given by the endogenous circulating oestrogen, will that not affect the quality of the life of the patient? Therefore, the ERα re-expression in ER-negative breast cancer cells for restoring response to endocrine therapy need to be thoroughly investigated using large cohorts of clinical trials.

As the mechanisms underlying endocrine resistance is very complex, for the benefit of these patients, exploring combination therapies are extremely important for improving the overall survival. Indeed, endocrine therapy combined with gefitinib, lapatinib or everolimus is currently under investigation in clinical trials. The study results have provided the evidence that combination therapy may improve the progression-free survival in treated patients [148,149]. A recent study also showed that gefitinib could reverse TAM resistance in breast cancer cells by inducing ERα re-expression [150]. The same group also previously showed that elemene (ELE), a traditional Chinese medicine, could reverse the TAM resistance of breast cancer cells and that ERα loss was the primary cause for the development of TAM resistance in these cells [151]. ELE appears to induce ERα re-expression by increasing the ERα transcript level to sensitize the cells to anti-oestrogens. It implies that re-exposure of ER-negative breast cancer patients to either drugs such as gefitinib, decitabine, ELE or LBH589 followed by endocrine therapy may benefit these patients and provide a novel therapeutic strategy for endocrine therapy. Although one such attempt was made, unfortunately, the clinical trial of combination therapy using tamoxifen in combination with decitabine, demethylating agents and LBH589, deacetylation inhibitor was discontinued. The reason being for early termination of the study was due to small numbers of participants analysed and technical problems.

## CONCLUDING REMARKS AND FUTURE PROSPECTS

Because endocrine resistance possesses a major challenge in treating the significant number of breast cancer cases, understanding the mechanisms that underlie the causes of this phenomenon is essential to reduce the burden of this disease. Although significant advancements are being made in the identification and characterization of several factors that contribute to the endocrine resistance, but our present understanding of this phenomenon is still at premature stage. Lack of ERα expression due to hypermethylation of *ESR1* promoter made researchers in this field to draw new strategies to re-express ERα in ER-negative breast cancers. Indeed, such strategies were successful in pre-clinical trials, but yet to reach the clinics. Of note, drugs such as SAHA in combination with herceptin perceived greater attention to show the promise in endocrine therapy [152]. Several miRNAs have been differentially expressed in endocrine cancers and emerged as new prognostic markers of the disease. More importantly, expression profiling studies showed overexpression of several ERα targeting miRNAs in ER-negative breast cancers suggesting that they can be served as bio-markers in the diagnosis and also in the management of breast cancer. Furthermore, developing the miRNA mimics as therapeutic drugs targeting these miRNAs will have the greater clinical value, but future awaits improving our technological advances in delivering these agents in the form of drugs into the sites of tumour. The other contributing factor for endocrine resistance is ERα-specific ubiquitin ligases. Because several lines of evidence suggest that re-expression of ERα in ER-negative breast cancer cells can restore sensitivity to tamoxifen, restoring the ERα expression by inhibiting ERα-specific Ub ligases provide potential novel strategies for restoring tamoxifen sensitivity. Therefore, small molecule inhibitors specific to these Ub ligases may overcome tamoxifen resistance in breast cancers. In particular, whether ER negativity is a cause or a consequence of the disease progression is a million dollar question in this field. Therefore, the debate continues until to unravel the precise mechanism(s) that explain the origin of ER negativity in breast cancer. Besides this, understanding tumour heterogeneity and real-time monitoring of early resistance to targeted therapies by analysing the resistant tumours through integrated approach is needed. We envisage more intensive research and debates with a resurgence of interest to better understand the ER negativity in breast cancer.

## Abbreviations

BL: basal-like
CBP: CAAT box-binding protein
CHIP: C-terminus of Hsp70-interacting protein
CSC: cancer stem cell
DNMT: DNA methyltransferase
EGFR: epidermal growth factor receptor
ELE: elemene
ERα: oestrogen receptor α
HDAC: histone deacetylase
Her-2: human epidermal growth factor receptor 2
IFI16: IFN-γ-inducible protein 16
Me: DNA methylation
MeCP2: methyl-CpG-binding protein 2
MTA: metastasis tumor antigen
MTOC: microtubule organizing center
NBL: non-basal-like
NuRD: nucleosome remodelling and deacetylase
p38MAPK: mitogen-activated protein kinase
PR: progesterone receptor
SAHA: suberoylanilide hydroxamic acid
SERM: selective oestrogen receptor modulator
TFAP2C: transcriptional factors AP-2γ
TNBC: triple-negative breast cancer
TSA: trichostatin A
VEGF: vascular endothelial growth factor
VHL: Von Hippel–Lindau
